# Screening Plants for Electrokinetic Petroleum Remediation: Tall Fescue Outperforms by Mitigating Soil Electrochemical Deterioration

**DOI:** 10.3390/plants15193075

**Published:** 2026-10-08

**Authors:** Peng Gao, Songyan Liu, Junhong Li, Yueyang Liu, Jiawei Jing

**Affiliations:** 1 Postdoctoral Station of Agricultural Resources and Environment, College of Land and Environment, Shenyang Agricultural University, Shenyang 110866, China; 2023500018@syau.edu.cn; 2 College of Engineering, Shenyang Agricultural University, Shenyang 110866, China; 18947579183@163.com (S.L.); 13317801592@163.com (J.L.); 2017500005@syau.edu.cn (Y.L.); 3 Key Laboratory of Arable Land Conservation (Northeast China), Ministry of Agriculture and Rural Affairs, Shenyang 110866, China; 4 National Engineering Research Center for Efficient Utilization of Soil and Fertilizer Resources, Shenyang 110866, China; 5 Shenyang Key Laboratory of Intelligent Manufacturing of Microbial Fertilizers, Shenyang 110866, China

**Keywords:** electrokinetic remediation, phytoremediation, plant screening, petroleum-contaminated soil, soil electrochemical properties, *Festuca arundinacea*

## Abstract

Electrokinetic (EK) remediation improves contaminant transport in low-permeability soils, but persistent electric fields degrade the soil electrochemical habitat, reducing treatment efficiency. Combining EK with plants may mitigate this deterioration, yet plant selection criteria remain unclear. This study screened tall fescue (*Festuca arundinacea*), ryegrass (*Lolium perenne*), corn grass (*Zea mexicana (Schrad.) Kuntze*), and alfalfa (*Medicago sativa*) in 60-day EK-assisted remediation of petroleum-contaminated silty clay under periodically reversed polarity. Petroleum removal, current dynamics, and soil physicochemical and biological indicators were monitored. Tall fescue performed best, achieving the highest petroleum removal (20.21%), greatest electrical conductivity, dissolved organic carbon, and field water-holding capacity, and the largest microbial abundance and dehydrogenase activity. Ryegrass performed similarly. Corn grass showed the tallest shoots but ranked third; alfalfa was least effective. Enhancement beyond simple additivity was species-dependent, observed only for tall fescue and ryegrass. Root-length stimulation correlated with remediation performance, while shoot-height response did not. Plants that mitigated electrochemical deterioration and sustained rhizosphere microbial activity were most suitable. Tall fescue is recommended, with ryegrass as a strong competitor. This study provides a transparent multi-indicator screening framework for selecting plants in EK-phytoremediation systems.

## 1. Introduction

Remediation of petroleum contamination in low-permeability soils is difficult because of limited hydraulic transport and low hydrocarbon bioavailability [1]. Electrokinetic (EK) remediation employs electromigration and electroosmosis to improve the movement of ions, water, and dissolved contaminants [2]. However, sustained EK application often disrupts the soil electrochemical environment, causing pH shifts, conductivity declines, colloid destabilization, and water redistribution, thereby progressively reducing treatment efficiency [3]. Phytoremediation depends on roots to release exudates, improve soil structure, and support hydrocarbon-degrading microorganisms, but is slow and diffusion-limited [4]. Therefore, combining these two approaches as EK-assisted phytoremediation holds significant promise, as the electric field would transport the contaminants and nutrients into the root zone, whereas plants will protect against the adverse electrochemical alterations that impair EK operation [5].

However, this combined performance strongly depends on selecting an appropriate plant species [6]. In the past, most studies investigated crude petroleum removal or the aboveground tolerance under conventional planting conditions, and plant performance under a continuous electric field remains elusive [7]. In an EK-assisted system, the plant should not just survive but actively stabilize the electrochemical conditions that maintain ion migration, current flow, and microbial metabolism. Furthermore, an efficient root system should buffer pH fluctuations, slow the loss of electrical conductivity (EC), stimulate microbial abundance and dehydrogenase activity, maintain field water-holding capacity, limit the colloid zeta potential increase, and provide dissolved organic carbon (DOC) [8]. Here, it was hypothesized that plants’ suitability for EK petroleum remediation is based on their ability to inhibit the deterioration of soil electrochemical conditions, such as pH shifts, conductivity loss, colloid destabilization, and water redistribution, rather than shoot vigor alone. This ability is likely associated with root-system traits [9]; however, its net effect can be assessed by different soil indicators.

To test this hypothesis, the following four plant species with different root architectures [10]: tall fescue (*Festuca arundinacea*), ryegrass (*Lolium perenne*), Mexican corn grass (*Zea mexicana (Schrad.) Kuntze*), and alfalfa (*Medicago sativa*) were analyzed in a 60-day EK-assisted remediation system. This study used a silty-clay soil that was artificially contaminated with crude oil. Furthermore, petroleum removal, current dynamics, and a set of biological and physicochemical indicators were assessed across plant-only, EK-only, and combined treatments. The findings indicated that tall fescue consistently outperformed other species, not by the largest shoot response but by the highest inhibition of soil electrochemical deterioration. This investigation aims to provide a screening technique and a theoretical explanation to develop more efficient and resilient EK-phytoremediation systems for petroleum-contaminated soils.

## 2. Results

### 2.1. Current Analysis and Degradation Rates

**Figure 1:**
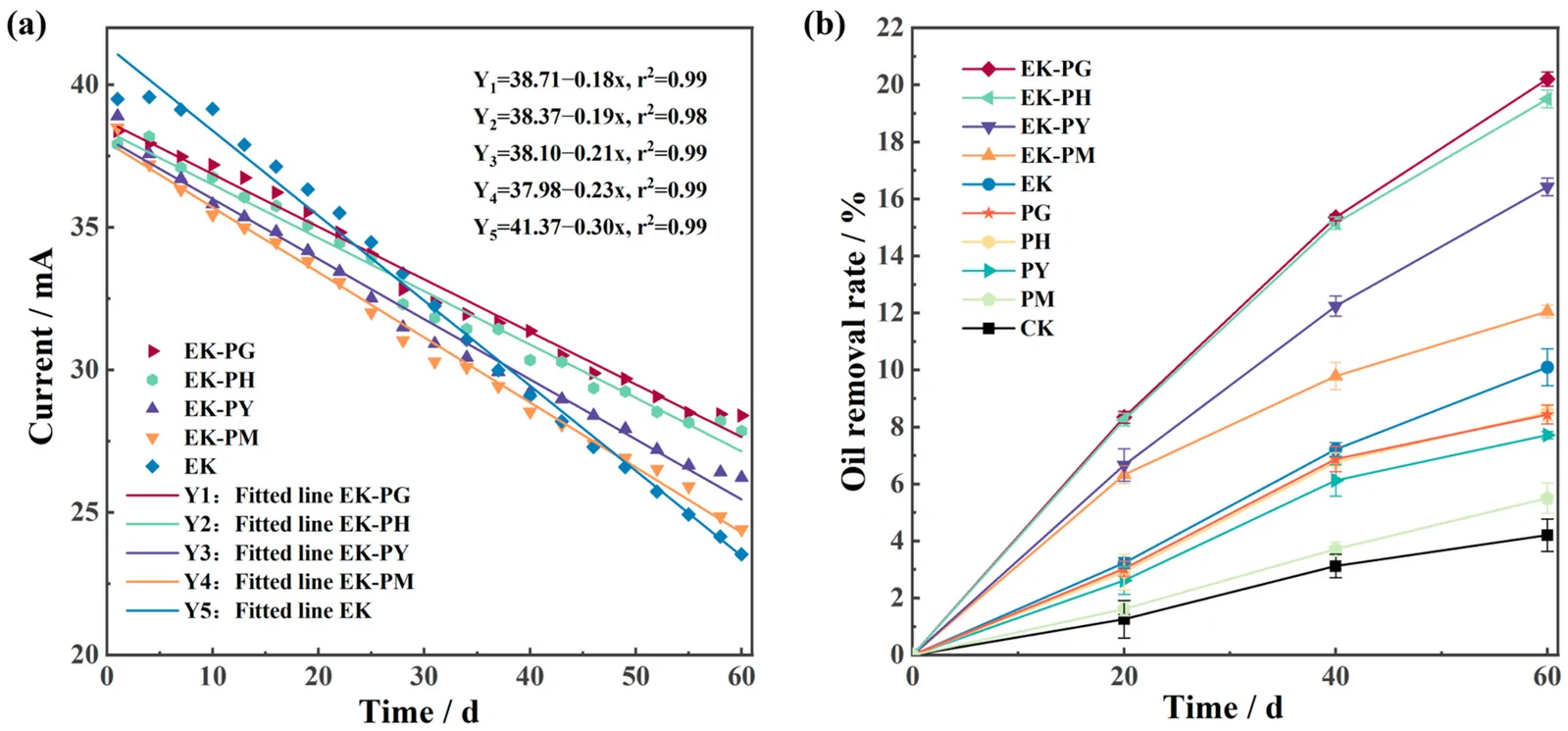
Current measurements (**a**) and degradation rates (**b**).

Current was constantly monitored in all EK treatments for 60 days (Figure 1a). At first mention, the treatment codes are defined as follows: EK-PG, EK combined with tall fescue; EK-PH, EK combined with ryegrass; EK-PY, EK combined with Mexican corn grass; EK-PM, EK combined with alfalfa; and EK, the plant-free electrokinetic treatment. The data revealed that the initial current values were between 37.9 and 39.5 mA and were highest in EK alone (39.5 mA). Furthermore, a decline was observed in each treatment, but the EK (39.90%) group day-60 decay was greater than that in EK-PG (27.97%), EK-PH (28.12%), EK-PY (32.60%), or EK-PM (36.62%) groups. The current retention ranked EK-PG ≈ EK-PH > EK-PY > EK-PM > EK, with the most stable currents observed in the two fibrous-rooted grasses.

After 60 days, petroleum removal ranked EK-PG (20.21 ± 0.25%) > EK-PH (19.50 ± 0.31%) > EK-PY (16.43 ± 0.31%) > EK-PM (12.06 ± 0.22%) (Figure 1b). For clarity, PG, PH, PY, and PM denote the corresponding plant-only treatments for tall fescue, ryegrass, Mexican corn grass, and alfalfa, respectively, whereas CK denotes the contaminated control treatment without plants or electrical treatment. Removal in EK, PG, PH, PY, PM, and CK was 10.10 ± 0.65%, 8.44 ± 0.33%, 8.51 ± 0.29%, 7.72 ± 0.13%, 5.51 ± 0.53%, and 4.21 ± 0.57%, respectively. The observed values in EK-PG (20.21%) and EK-PH (19.50%) exceeded the uncorrected arithmetic sums of the corresponding EK-only and plant-only removals (18.54% and 18.61%, respectively), whereas EK-PY (16.43% vs. 17.82%) and EK-PM (12.06% vs. 15.61%) did not. Because CK showed 4.21% background loss, these arithmetic comparisons are descriptive and are not treated as a formal statistical test of synergy. The parallel rankings of removal and current retention suggest an association between sustained EK transport and remediation performance [11].

### 2.2. Soil Physicochemical Properties

At day 60, colloid zeta potential, EC, pH, field water-holding capacity, and DOC were assessed to identify electric-field-mediated deterioration and plant-induced mitigation. The data revealed that at day 60, pH was 7.52 ± 0.19 in CK and 7.15 ± 0.11 (PH) to 7.36 ± 0.11 (PM) in plant-only treatments (Table S2). Furthermore, EK induced minimal net pH change: 7.47 ± 0.13 in the electrode region and 7.50 ± 0.12 elsewhere, which aligns with hourly polarity reversal minimizing extreme gradients [12]. Electrode/non-electrode pH values were 7.15 ± 0.15/7.12 ± 0.15 (EK-PH), 7.11 ± 0.11/7.13 ± 0.14 (EK-PG), 7.30 ± 0.21/7.27 ± 0.16 (EK-PY), and 7.37 ± 0.23/7.33 ± 0.16 (EK-PM). Of these treatment groups, EK-PG and EK-PH had the closest values to circumneutral pH, whereas EK-PM demonstrated minimal alterations from EK.

**Figure 2:**
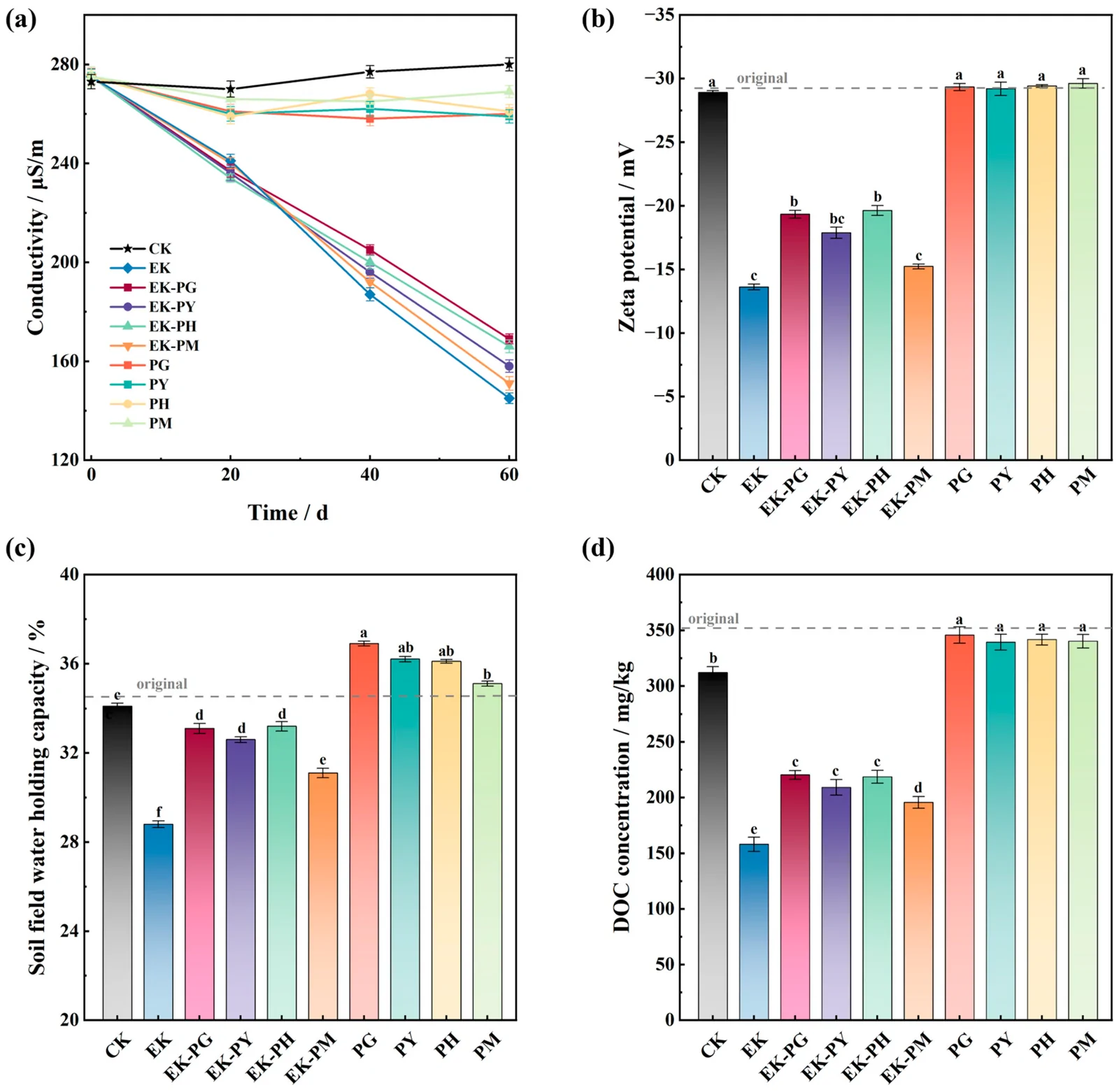
Soil physicochemical characteristics of different treatments at day 60. (**a**) Electrical conductivity dynamics over 60 days. (**b**) Soil colloid zeta potential at day 60. (**c**) Field water-holding capacity at day 60. (**d**) Dissolved organic carbon content at day 60.

This study also showed that at day 60, EC was reduced in all treatment groups from 275 µS/cm, indicating the highest decrease in the EK group (145 ± 2.11 µS/cm) (Figure 2a). The reduced EC values were 169 ± 1.99 (EK-PG), 166 ± 2.56 (EK-PH), 158 ± 2.52 (EK-PY), and 151 ± 2.74 µS/cm (EK-PM), compared to 259 ± 2.71 to 269 ± 2.42 µS/cm in plant-only controls. Therefore, the EC retention in different treatment groups was ranked as follows: EK-PG > EK-PH > EK-PY > EK-PM > EK, consistent with current retention.

Moreover, the initial zeta potential was −29.27 mV. At day 60, the zeta potential in EK reached −13.61 ± 0.22 mV, corresponding to a 53.47% shift toward zero and indicating the greatest colloid destabilization (Figure 2b). This shift was attenuated by planting: −19.33 ± 0.31 mV in EK-PG (33.96% shift toward zero), −17.87 ± 0.44 mV in EK-PY (38.95%), −19.62 ± 0.39 mV in EK-PH (32.97%), and −15.22 ± 0.19 mV in EK-PM (48.00%). Plant-only values remained close to baseline (−29.19 ± 0.52 to −29.61 ± 0.37 mV).

Initially, the field water-holding capacity was 30.5%, but at day 60, this value reached 28.8 ± 0.15% in EK, 35.1 ± 0.11% to 36.9 ± 0.11% in plant-only treatments, and 33.1 ± 0.23%, 33.2 ± 0.21%, 32.6 ± 0.13%, and 31.1 ± 0.21% in EK-PG, EK-PH, EK-PY, and EK-PM, respectively (Figure 2c). It was inferred that under the electric field, the water-holding capacity was best preserved in fibrous-rooted grasses.

DOC analysis revealed that by day 60, DOC values were highest in plant-only treatments (339.2 ± 7.15 to 345.6 ± 7.33 mg/kg), 312 ± 5.31 mg/kg in CK, and lowest in EK (158 ± 6.36 mg/kg) (Figure 2d). The decrease in EK might reflect electrochemical oxidation and electroosmotic flushing of soluble organics [13]. The DOC content of EK-PG, EK-PH, EK-PY, and EK-PM was 220.3 ± 3.94, 218.6 ± 5.78, 209.1 ± 7.01, and 195.6 ± 5.33 mg/kg, respectively, indicating the highest reduction by tall fescue and ryegrass.

Across the five physicochemical indicators, EK alone showed the greatest deterioration, whereas plants reduced colloid destabilization, conductivity loss, loss of water-holding capacity, and DOC depletion. The most consistent protection was provided by tall fescue and ryegrass, followed by Mexican corn grass and alfalfa.

### 2.3. Biological Characteristics

**Figure 3:**
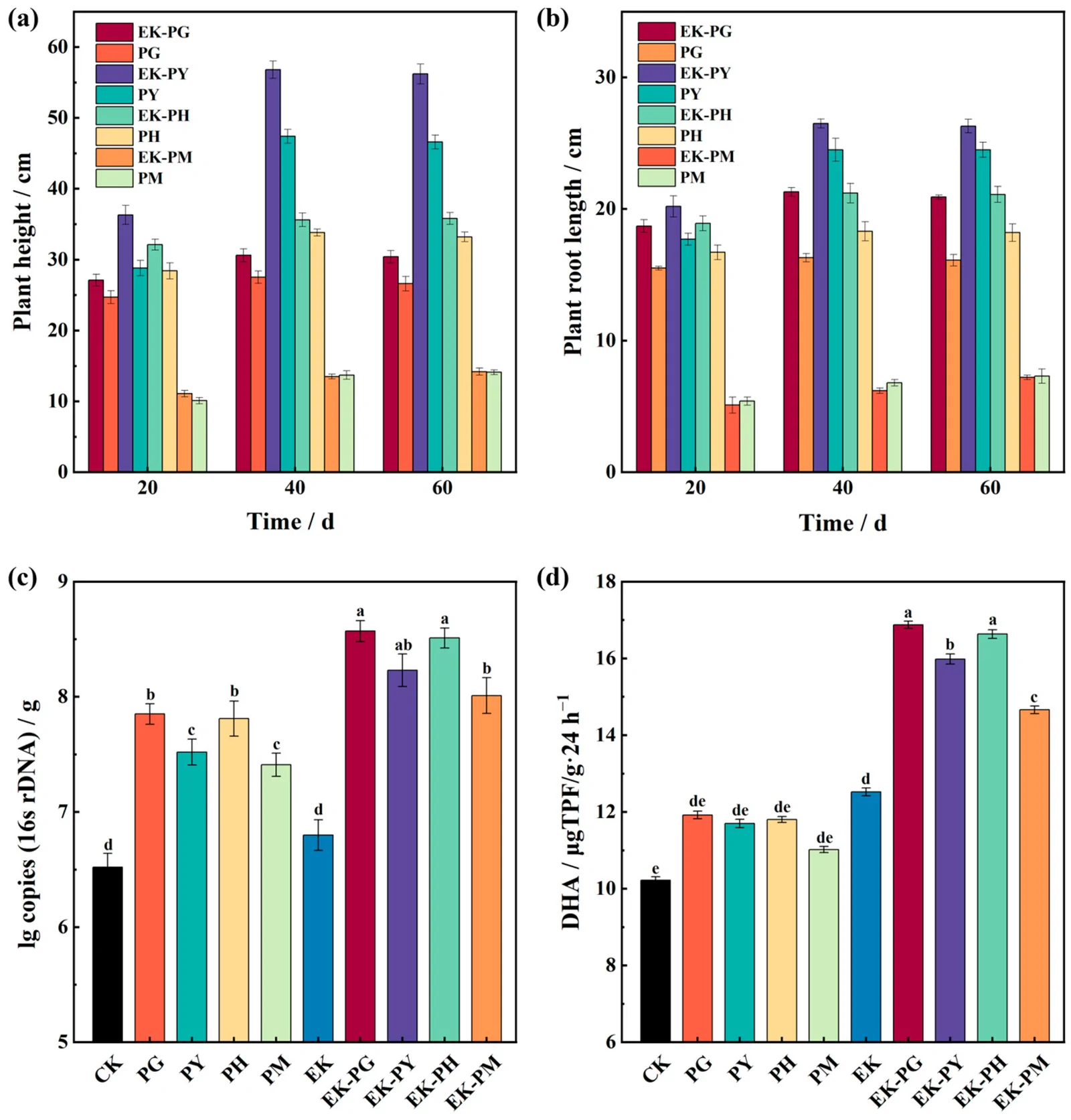
Biological characteristics of different treatments. (**a**) Shoot height and (**b**) root length at days 20, 40, and 60. (**c**) Microbial abundance (16S rRNA gene copy number) and (**d**) dehydrogenase activity at day 60.

To compare species responses and their association with remediation performance, plant growth and soil biological activity were assessed at days 20, 40, and 60. The electric field induced shoot elongation in all species, with species-dependent magnitudes [14]. At day 60, shoot-height alterations compared to plant-only controls were 14.29% in tall fescue (30.4 ± 0.88 vs. 26.6 ± 1.03 cm), 7.83% in ryegrass (35.8 ± 0.84 vs. 33.2 ± 0.68 cm), 20.60% in corn grass (56.2 ± 1.41 vs. 46.6 ± 0.99 cm), and 0.71% in alfalfa (14.2 ± 0.51 vs. 14.1 ± 0.33 cm) (Figure 3a,b). Corn grass demonstrated the tallest shoots and the highest relative increase.

Root responses differed among treatments (Figure 3b). At day 60, root length relative to plant-only controls changed by +29.81% in tall fescue (20.9 ± 0.16 vs. 16.1 ± 0.44 cm), +15.93% in ryegrass (21.1 ± 0.61 vs. 18.2 ± 0.66 cm), +7.35% in Mexican corn grass (26.3 ± 0.52 vs. 24.5 ± 0.57 cm), and −1.37% in alfalfa (7.2 ± 0.55 vs. 7.3 ± 0.17 cm). The root-length response therefore ranked EK-PG > EK-PH > EK-PY > EK-PM, consistent with current retention and petroleum removal but not with shoot-height response (EK-PY > EK-PG > EK-PH > EK-PM).

At day 60, bacterial abundance was lowest in CK (lg 6.52 ± 0.12 copies/g), ranging between lg 7.41 ± 0.10 (PM) and lg 7.85 ± 0.09 (PG) copies/g in plant-only treatments, and was lg 6.80 ± 0.13 copies/g in EK (Figure 3c). Moreover, the bacterial abundance in EK–plant treatments was highest: lg 8.57 ± 0.09 (EK-PG), lg 8.51 ± 0.09 (EK-PH), lg 8.23 ± 0.14 (EK-PY), and lg 8.01 ± 0.16 (EK-PM) copies/g, indicating an increase of 31.44%, 30.52%, 26.23%, and 22.85%, respectively compared to CK. This ranking aligned with petroleum removal.

The dehydrogenase activity at day 60 was 11.02 ± 0.08 to 11.92 ± 0.10 μg TPF/g/24 h in plant-only treatments, 10.22 ± 0.09 μg TPF/g/24 h in CK, and 12.53 ± 0.10 μg TPF/g/24 h in EK (Figure 3d). This activity was highest in EK-PG (16.88 ± 0.10), EK-PH (16.63 ± 0.11), EK-PY (15.98 ± 0.13), and EK-PM (14.66 ± 0.10 μg TPF/g/24 h), similar to the order observed for bacterial abundance and removal.

The results revealed that EK–plant combinations increased bacterial abundance and dehydrogenase activity relative to either EK or plant alone. Species with the strongest root-length and microbial responses (tall fescue and ryegrass) also indicated the highest petroleum removal, followed by corn grass, which ranked third despite its superior shoot response.

### 2.4. Integrated Comparison for Plant Selection

**Figure 4:**
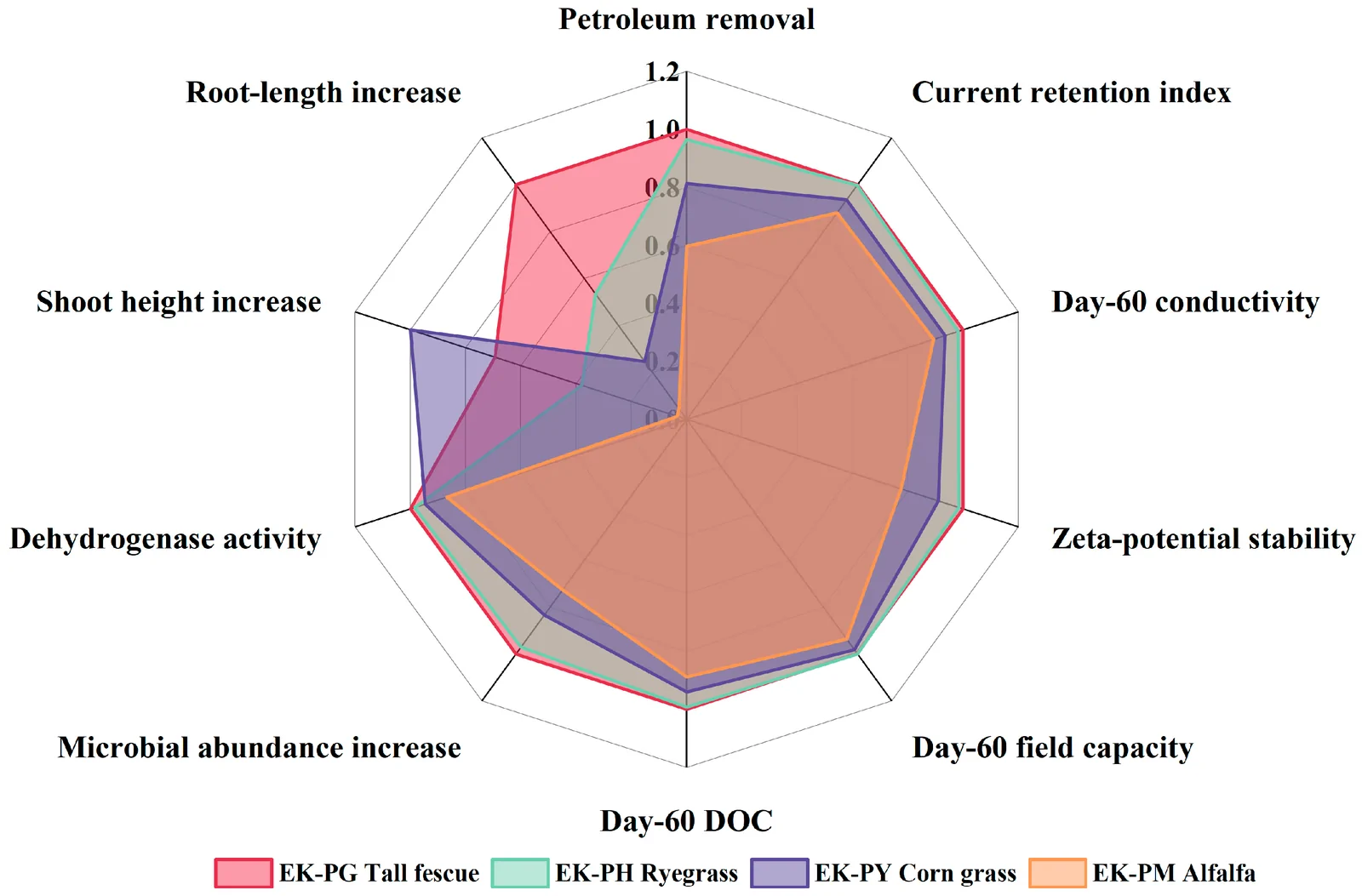
Radar chart comparing the integrated performance of the four EK-plant treatments at day 60. Axes indicate 10 measured indicators covering physicochemical, electrochemical, and biological dimensions. Increased current decay and zeta-potential (negative indicators, with lower values indicating better performance) were positively transformed into current retention index (100% − decay) and zeta-potential stability (100% − increase), respectively.

For integrated species selection, the following 10 variables were displayed in a radar chart (Figure 4): petroleum removal, day-60 EC, current retention, zeta-potential stability, day-60 field water-holding capacity, bacterial-abundance increase relative to CK, day-60 DOC, dehydrogenase activity, and alterations in shoot-height and root-length relative to plant-only controls. The zeta-potential stability and current retention were calculated as 100% minus current decay and zeta-potential increase, respectively; each indicator was normalized to its maximum treatment mean.

Tall fescue and ryegrass yielded broad radar profiles, with tall fescue indicating highest values on eight of ten indices, including removal, EC, zeta-potential stability, DOC, current retention, bacterial abundance, dehydrogenase activity, and root stimulation (Figure 4). Furthermore, ryegrass showed similar data for EC, zeta potential, DOC, and bacterial abundance, but slightly higher values for water-holding capacity, while demonstrating weaker root and shoot responses. Corn grass showed the greatest shoot response but ranked third for current retention, removal, EC, and bacterial abundance and scored < 0.95 on all other axes. Alfalfa scored < 0.90 on eight indices and demonstrated negligible shoot and root stimulation.

For tall fescue, ryegrass, corn grass, and alfalfa, the equal-weight selection index, based on indicators normalized to the maximum among treatments, was 96.9, 87.9, 85.4, and 66.5, respectively. Therefore, the plant species were ranked in the following order: *Festuca arundinacea* > *Lolium perenne* > *Zea mexicana (Schrad.) Kuntze* > *Medicago sativa*, which aligns with Section 3.1, Section 3.2 and Section 3.3. Tall fescue had a wider index advantage over ryegrass, reflecting modest gains across multiple indicators, particularly its nearly twofold greater root-length stimulation.

The integrated analysis revealed that plant selection cannot be based on a single trait, specifically shoot-height response. Tall fescue yielded better results because it integrated consistently high physicochemical, electrochemical, and biological performance rather than producing the tallest shoots.

## 3. Discussion

### 3.1. Plant Mitigation of Electrochemical Deterioration

This study observed that plants consistently reduced electric-field-mediated deterioration, but in a species-dependent manner. In EK alone, zeta potential shifted > 50% toward zero, EC was reduced by nearly half, field water-holding capacity declined below baseline, and DOC decreased to about half the CK value. These alterations were consistent with ion redistribution, electrolysis-related pH shifts, electroosmotic water loss, and electrochemical oxidation of organic matter in EK systems [15], which gradually disrupts the soil habitat and can limit prolonged treatment.

All planted EK treatments attenuated this deterioration. Tall fescue and ryegrass plants indicated the highest EC, water-holding capacity, and DOC, as well as the most negative zeta potentials, whereas corn grass plants were ranked intermediate and alfalfa least effective. These species showed consistent ranking in all indicators, and petroleum removal was associated with soil physicochemical quality preservation and remediation performance.

Although this study did not directly quantify root architecture, the pattern was compatible with reported species differences. Tall fescue develops a dense fibrous root system [16], alfalfa has a coarser taproot with reduced surface-soil root-length density [17], and ryegrass indicated a generally less dense fibrous system than tall fescue [18]. Tall fescue demonstrated the highest root-length stimulation (+29.81%). Roots comprise water-filled conductive pores [19]; mucilage and exudates can buffer pH, complex ions, and stabilize colloids [20]; and rhizospheres can retain organic carbon and water during EK depletion and drying [21]. Therefore, a well-developed root system may stabilize the electrochemical habitat; however, direct root-trait analyses are warranted to confirm this mechanism.

### 3.2. Decoupling of Aboveground Tissue Growth and Remediation Performance

Aboveground tissue growth, as represented here by shoot height, was decoupled from remediation performance. Although Mexican corn grass showed the greatest shoot-height increase (+20.60%) and the tallest shoots, it ranked third for current retention, petroleum removal, and microbial activity. Tall fescue had a smaller shoot-height response (+14.29%) but ranked first for the principal remediation indicators. Thus, shoot-height response alone is insufficient for selecting plants for EK-assisted phytoremediation [22]. Because leaf area, photosynthetic rate, chlorophyll content, phenolic content, and other physiological stress indicators were not reported in this study, the present data should not be interpreted as a comprehensive assessment of plant stress tolerance.

Shoot-height response was decoupled from remediation performance. Although corn grass showed the greatest height increase (+20.60%) and the tallest shoots, it was ranked third for current retention, petroleum removal, and microbial activity. Tall fescue had a smaller shoot response (+14.29%), but was ranked first for all remediation-critical indicators. Therefore, apparent stress tolerance or shoot-height response alone is insufficient for selecting plants for EK-assisted phytoremediation [22].

Species also indicated differences in shoot and root allocation. The root length of corn grass increased by only 7.35%, whereas that of tall fescue increased by 29.81%, indicating the largest response. This association suggests that under EK conditions, plant species favoring belowground may be better remediation partners [8]. Possible drivers include altered nutrient gradients, electrotropism, and species-specific hormonal responses; however, these mechanisms have not been investigated [23,24].

### 3.3. Species-Dependent Synergistic Enhancement of Biological Activity and Hydrocarbon Removal

Petroleum removal was substantially enhanced when EK remediation was combined with plants, and the magnitude of this enhancement was species-dependent. Importantly, the synergistic effect should not be interpreted as the simple arithmetic sum of petroleum removal achieved by EK alone and plant treatment alone, because both individual treatments include the background petroleum loss observed in CK. Therefore, the expected additive removal was estimated after correcting for background loss as:

*RE_additive_* = *RE_EK_* + *RE_plant_* − *RE_CK_* (1)

where *RE* denotes petroleum removal efficiency.

Based on this background-corrected expectation, the predicted additive removals were 14.33%, 14.40%, 13.61%, and 11.40% for tall fescue, ryegrass, corn grass, and alfalfa, respectively, whereas the corresponding observed removals in the combined EK–plant treatments reached 20.21%, 19.50%, 16.43%, and 12.06%. Thus, the combined treatments exceeded the background-corrected additive expectation by 5.88, 5.10, 2.82, and 0.66 percentage points, respectively. These results demonstrate a positive interaction between EK and phytoremediation rather than a simple superposition of their individual effects, with the synergistic enhancement being strongest for tall fescue and ryegrass. Therefore, the extent of synergy depended strongly on plant species and their compatibility with the electrokinetic environment [25].

The mechanistic context for this species dependence can be explained by the biological data. The EK–plant treatments indicated the highest microbial abundance and dehydrogenase activity, with tall fescue and ryegrass favoring the largest and most active communities. Furthermore, EK alone only yielded a modest increase, aligning with the findings that electrochemical stress can inhibit sensitive populations [13]. Plants may develop rhizosphere microenvironments against the harshest electrochemical conditions, while benefiting from EK-induced nutrients and electron acceptor transport [26]. Low-molecular-weight compounds released by roots can act as biosurfactants or co-substrates, elevating hydrocarbon bioavailability and inducing co-metabolic degradation [17,27]. This mechanism is supported by the higher DOC in planted treatments. Microbial activity, DOC, and petroleum removal indicated the following rank order: EK-PG > EK-PH > EK-PY > EK-PM.

Compared to plant-only controls, the EK–plant treatments showed increased dehydrogenase activity relative to bacterial abundance. This pattern is consistent with increased community-level metabolic activity by improved nutrient supply or contaminant accessibility; however, it cannot explain higher activity for independent cells [28,29]. However, greater abundance and oxidative activity both support a biological contribution to hydrocarbon removal.

### 3.4. Limitations and Implications for Plant Selection

This study has several limitations. (1) The root density and architectural traits were not directly evaluated; therefore, the association between root development and electrochemical stabilization was inferred from root-length data and the literature on the root systems of the tested species. Therefore, future studies should perform quantitative analyses of specific root length, root length density, root surface area, or root diameter distribution to directly evaluate the hypothesized root-architecture mechanism [26]. (2) Plant physiological stress was not comprehensively evaluated in this study. Shoot height and root length were used as growth-response indicators, whereas biomass, leaf area, photosynthetic activity, chlorophyll content, and other physiological parameters were not measured. Future studies should incorporate these indicators to provide a more comprehensive assessment of plant responses to electrokinetic conditions [30]. (3) Functional gene abundance and microbial community composition were not characterized, and although the increases in dehydrogenase activity and 16S rRNA gene copy number reflect greater oxidative metabolism and overall microbial biomass, the hydrocarbon-degrading populations and associated metabolic pathways remain undetermined [31]. Therefore, amplicon sequencing or metagenomic analysis should be conducted to add valuable mechanistic resolution [32]. (4) The study was carried out at pot scale (24 × 15 × 16 cm) with one voltage gradient, a single crude-oil concentration (3% *w*/*w*), and a fixed polarity-reversal schedule (1 h). Field conditions, including voltage gradients, salinity, soil texture, hydrocarbon composition, and climatic factors, vary widely and warrant further validation at mesocosm or field scale [33]. (5) The 60-day duration may not completely indicate longer-term dynamics, such as progressive root-system development, potential plant adaptation, or late-stage electrochemical alterations [34].

Despite these limitations, the study provides a practical framework for plant selection. Suitable plants should not only exhibit favorable aboveground tissue growth but also mitigate electrochemical habitat disruption and sustain rhizosphere microbial activity. This function can be assessed by measuring EC, bacterial abundance, field water-holding capacity, zeta potential, DOC, and dehydrogenase activity, which together characterize soil functional quality under an applied electric field. Tall fescue showed the most balanced and consistently high performance and was therefore selected for subsequent EK-assisted petroleum-remediation analysis. Ryegrass showed a similar pattern and represents the strongest comparison candidate. Future work should validate both species at mesocosm or field scale across variable voltage gradients, salinity conditions, soil textures, hydrocarbon loads, and climatic regimes, together with microbial-community and functional-gene analyses, direct root-trait measurements, comprehensive plant-stress indicators, and life-cycle cost assessment [35].

## 4. Materials and Methods

### 4.1. Soil, Contaminant, and Contaminated-Soil Preparation

Surface soil (0–20 cm) was sampled from farmland in northern Shenyang, China. Then, plant residues and debris were removed, and the soil was air-dried and filtered through a 2 mm sieve. The silty clay comprised clay (24.1%), silt (60.2%), and sand (15.7%), with an initial pH of 7.69, 19.8 g/kg organic matter, 0.248 mS/cm EC, and 30.5% field water-holding capacity. Furthermore, the soil phosphorus and potassium content were 23.5 and 52.9 mg/kg, respectively (Table S1). Crude oil was acquired from the Liaohe Oilfield and had a density of 0.903 g/cm^3^ at 25 °C, a solidification point of 26.0 °C, and contained 36.22% aromatics, 25.73% alkanes, 18.28% resins, and 19.77% asphaltenes. Crude oil was first dissolved in chloroform to facilitate its uniform distribution in the soil. The resulting oil solution was thoroughly mixed with a small portion of the clean, sieved soil to prepare a high-concentration petroleum-contaminated stock soil. This stock soil was then progressively mixed with the remaining clean soil until a homogeneous mixture was obtained, resulting in an artificially contaminated soil with a final petroleum concentration of 3% (*w*/*w*) [36]. The contaminated soil was stirred twice daily in a fume hood until the chloroform had completely evaporated. The homogeneity of petroleum distribution was subsequently verified by randomly sampling the prepared soil for petroleum analysis, after which the contaminated soil was aged for 30 days [11].

### 4.2. Plant Materials and Experimental Design

This study analyzed the following four plant species with contrasting root characteristics: tall fescue (*Festuca arundinacea*, PG), ryegrass (*Lolium perenne*, PH), Mexican corn grass (*Zea mexicana (Schrad.) Kuntze*, PY), and alfalfa (*Medicago sativa*, PM). Briefly, plant seeds were surface-sterilized with a 20% (*v*/*v*) commercial sodium hypochlorite solution for 10 min, washed five times with sterile water, soaked for 24 h, germinated at 25 °C, and then uniform seedlings were transplanted [37].

This experiment comprised 10 treatments, each treatment was established in three independent replicate units (*n* = 3). (Table S2): a no-EK control (CK; contaminated and unplanted), EK without plants (EK), 4 EK–plant treatments (EK-PG, EK-PH, EK-PY, and EK-PM), and 4 plant-only treatments (PG, PH, PY, and PM). The seedlings were planted at 1 plant·cm^−2^. Plant and soil sampling was conducted at days 20, 40, and 60. For destructive plant measurements, different individuals were randomly selected at each sampling time, and harvested plants were not reused in subsequent evaluations. Owing to the planting density of 1 plant·cm^−2^, sufficient plants remained in each experimental unit for successive destructive sampling. Soil samples were collected from predefined locations within each replicate, with only the amount required for the corresponding analyses being removed at each sampling event. Before loading, deionized water was added to the contaminated soil to 70% of field water-holding capacity.

### 4.3. EK Setup and Operating Conditions

**Figure 5:**
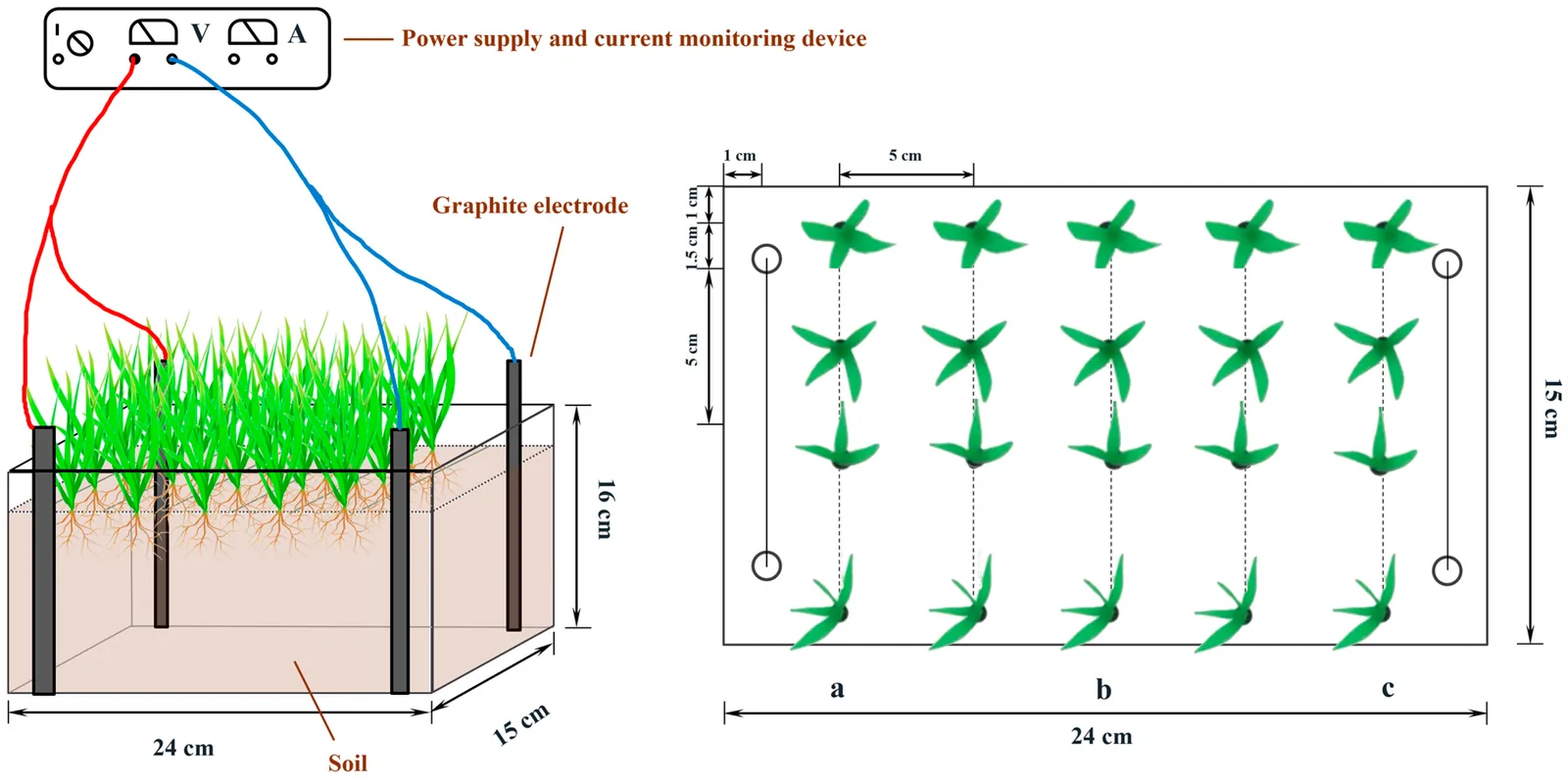
Schematic illustration of the EK-assisted phytoremediation system. Note: this figure is intended only to illustrate the experimental setup and is not drawn to scale. The number and spacing of seedlings shown do not represent the actual planting density. The actual planting density used in the experiment was 1 plant·cm^−2^. Electrode region indicates sampling positions adjacent to the graphite electrodes (a, c); non-electrode region refers to the central sampling position (b).

The apparatus comprised a 24 V DC power supply, real-time current monitoring, a soil chamber (24 × 15 × 16 cm), two graphite–electrode pairs (15 × 0.8 cm) at opposite chamber ends, and an automatic polarity-reversal controller (Figure 5). A voltage gradient of 1 V·cm^−1^ was selected with reference to previous electrokinetic–phytoremediation studies, in which low-voltage electric fields around this level were considered suitable for promoting electrokinetic transport while maintaining plant compatibility [38]. Periodic polarity reversal was employed to mitigate the adverse effects of prolonged unidirectional electrokinetic treatment on soil physicochemical conditions, including electrode polarization and the development of localized pH gradients [12]. The plants were maintained in a greenhouse at 25 ± 2 °C with a 16 h/8 h light/dark cycle. Every 3 d, deionized water was added at the same time to maintain comparable moisture, and treatment positions were rotated to limit the effects of light and temperature gradients.

### 4.4. Sampling and Analytical Methods

Soil and plants were sampled every 20 d. From each predefined point, soil (10 g) was collected, and samples along each longitudinal line were composited. Soil pH was measured in a 1:2.5 (*w*/*v*) soil–water suspension [39]. EC was determined in the aqueous extract at a soil-to-water ratio of 1:5 (*w*/*v*) after shaking with ultrapure water, and measured with a calibrated digital conductivity meter (DDS-307A, INESA Scientific Instrument Co., Ltd., Shanghai, China) [40]. Field water-holding capacity was measured by the ring-knife method [41]. Soil colloid zeta potential was determined from the supernatant of a sieved (<100 mesh) soil suspension in deionized water after ultrasonication and settling [42]. DOC was extracted by hot water and quantified using a total organic carbon analyzer [43]. Soil organic matter was determined by dichromate oxidation titration [44]. Petroleum was ultrasonically extracted with dichloromethane, centrifuged, filtered through anhydrous sodium sulfate, and quantified gravimetrically after solvent evaporation [45]. Removal was calculated as the percentage decrease from the initial concentration [46]. Shoot height and root length were measured for 20 randomly selected plants per treatment; biomass was dried at 85 °C to constant weight. Microbial abundance was quantified by real-time quantitative PCR (qPCR) targeting the bacterial 16S rRNA gene [47]. Soil dehydrogenase activity was determined using the triphenyl tetrazolium chloride reduction method [48].

### 4.5. Integrated Selection Index

To compare the overall performance of the four plant species, ten indicators covering electrochemical, physicochemical, and biological dimensions were integrated into a single selection index. Two indicators—current decay and zeta-potential increase—were negative (lower values indicating better performance) and were first transformed into positive indicators:

Current retention index = 100% − current decay (2)

Zeta-potential stability = 100% − zeta-potential increase (3)

Each indicator was then normalized to the maximum value among the four electrokinetic-plant treatments:

(4) $$x_{\mathrm{norm},i}=\frac{x_{i}}{x_{\max}}$$

where *x_i_* is the value of the indicator for treatment *i*, and *x_max_* is the maximum value of that indicator among the four treatments. This normalization scales each indicator to a range of 0–1.

The integrated selection index was calculated as the equal-weight average of the ten normalized indicators, multiplied by 100:

(5) $$\mathrm{Selection}\;\mathrm{index}=\frac{1}{N}\sum_{j=1}^{N}x_{\mathrm{norm},j}\times 100$$

where *N* = 10 is the number of indicators. Equal weighting was applied because no prior basis existed for assigning differential importance among the ten indicators.

### 4.6. Statistical Analysis

All assessments were carried out in triplicate, and values are expressed as mean ± standard deviation. Statistical differences among treatments were evaluated by one-way analysis of variance (ANOVA) with Tukey’s HSD post hoc test using SPSS 21.0. Differences were considered significant at *p* < 0.05.

## 5. Conclusions

All four planted EK treatments reduced current decay and increased petroleum removal compared to the EK-only treatment, but there were substantial species-related differences. Tall fescue (*Festuca arundinacea*) indicated the best overall performance, with the highest petroleum removal (20.21%), microbial abundance, current retention, and dehydrogenase activity, and the most efficient reduction in conductivity loss and colloid destabilization. Ryegrass (*Lolium perenne*) performed nearly as well and is considered a strong comparative candidate. Despite the largest shoot-height response, corn grass (*Zea mexicana (Schrad.) Kuntze*) ranked third, validating that shoot-height response is an unreliable selection criterion. Alfalfa (*Medicago sativa*) was the least efficient partner. These results indicated that plants that can inhibit electrochemical deterioration and sustain microbial activity are suitable for EK-assisted remediation; these abilities are likely related to root-system development, though direct quantification requires further study. This multi-indicator framework provides a transparent basis for selecting species and can help develop more effective EK-phytoremediation systems.

## Data Availability

The original contributions presented in this study are included in the article material. Further inquiries can be directed to the corresponding author.

## Supplementary Materials

The following supporting information can be downloaded at: https://www.mdpi.com/article/10.3390/plants15193075/s1, Table S1: The initial characteristics of sampled soil; Table S2: Soil pH in different treatments at day 60.

## Abbreviations

The following abbreviations are used in this manuscript:

EK	Electrokinetic
DOC	Dissolved organic carbon
