# Supplementary material for: Screening Plants for Electrokinetic Petroleum Remediation: Tall Fescue Outperforms by Mitigating Soil Electrochemical Deterioration
Source: Plants (Basel). 2026 Oct 8;15(19):3075. doi: 10.3390/plants15193075 (PMC13666624; doi:10.3390/plants15193075)
Supplement: Supplementary file 1 [file plants-15-03075-s001.zip › plants-4535590-supplementary.pdf]

Table S1: The initial characteristics of sampled soil.

Soil properties		value
pH		7.69
Organic matter ($g \cdot kg^{-1}$)		19.8
Conductivity ($mS \cdot cm^{-1}$)		0.248
	< 0.002	24.1
Soil Texture (mm, %)	0.002- 0.02	60.2
	0.02- 2	15.7
Available phosphorus ($mg \cdot kg^{-1}$)		23.5
available potassium ($mg \cdot kg^{-1}$)		52.9
Soil field capacity (%)		34.5

Table S2 Soil pH in different treatments at day 60.

Group	Electrode region	Non-electrode region
CK	—	7.52 ± 0.19
PG	—	7.21 ± 0.11
PY	—	7.34 ± 0.11
PH	—	7.15 ± 0.11
PM	—	7.36 ± 0.11
EK	7.47 ± 0.13	7.50 ± 0.12
EP-PM	7.37 ± 0.23	7.33 ± 0.16
EP-PG	7.11 ± 0.11	7.13 ± 0.14
EP-PH	7.15 ± 0.15	7.12 ± 0.15
EP-PY	7.30 ± 0.21	7.27 ± 0.16

Note: Electrode region indicates sampling positions adjacent to the graphite electrodes (a, c); non-electrode region refers to the central sampling position (b).
